# The Homœopathic Medical Society of Central New York

**Published:** 1882-03

**Authors:** C. P. Jennings

**Affiliations:** Secretary


### THE CENTRAL NEW YORK HOMŒOPATHIC MEDICAL SOCIETY.

The society met at Syracuse, Dec. 15th, 1881, the President in the chair. A discussion on paragraph 185 of the Organon was opened by Dr. Boyce, who said that no remedy was given internally without some effect; the system responds immediately, and even dynamic medicines affect many in health; some persons being very susceptible to their influence. We do not yet know whether or not dilutions, in a single dose, will produce pathogenetic effects on the healthy.

Dr. Nash: Effects may be present without being perceptible.

Dr. Seward: Some are only affected by repetition.

Dr. Boyce: It may require idiosyncrasy to secure an effect in a well man; but there are persons who are affected. The term energetic is applicable to any medicine. Some persons are affected by only certain medicines, while others again are not in the least influenced by them; but other medicines will be found to have an effect on these.

Dr. Nash then wished to know if alternation could be recommended.

Dr. Seward: Suggested that it was only necessary to wait till the remedy given had exhausted its power, and that if another remedy was necessary it should succeed the first, in succession, and not alternation.

Dr. Boyce: A single dose must expend itself, and the case be again studied to see what is necessary. This is Homœopathy. If satisfied you have made a mistake, change. You can easily derange a case by repeating the appropriate remedy.

Dr Nash related a case of pneumonia in which a dry dose had no apparent effect, he then repeated the dose in twenty-four hours, and still no effect; after dissolving the same remedy in water, and giving a dose every two hours, there was a good response. He then asked, how was it cures followed repetition of the dose, notwithstanding one dose produced effect? Some are in the habit of giving medicine in solution till they have a result, and then discontinue. What danger is there in repeating a remedy before the first dose is exhausted.

Dr. Boyce: Take *Aconite* for example: A dose produces an effect; you give another dose just when, perhaps, reaction is setting in, and you keep on giving until the case is so confused you can make nothing of it. Dr. Dunham instructed me to wait on the one dose, yet I read of him giving *Lachesis* in carbuncle repeated every day. What ground can I stand upon? I had a severe case of diphtheria which agitated me for fear of a fatal result; single doses brought the patient through safely. *Belladonna* being indicated the single dose produced a good result. When the deposit showed itself one dose of the appropriate remedy was given and not repeated for forty-eight hours; generally the case was thus mitigated. Giving one dose and waiting has been more successful than frequent repetition.

Dr. Nash: The primary effect of a drug is always manifest before the secondary; if we push the medicine, so as to overcome reaction, we do mischief; we may repeat without overcoming reaction, if it is too strong to be overcome.

Dr. Boyce: When will you repeat the dose? How are you going to tell when the medicine shall be repeated?

Dr. Nash: Each case must be prescribed for by itself, according to its own indications. There cannot be any rule for repeating after a certain number of hours.

Dr. Seward asked if he would depend on one dose in cholera.

Dr. Nash replied, yes, if the one dose started a decided improvement.

Dr. Seward: When there is little power to react, and the disease severe, then would not frequent repetition be needed to get up reaction?

Dr. Boyce: Just what we want is this,—we are satisfied with the law of similars and the potencies—but how are we to know when to repeat our doses? Hahnemann says (p. 152, Chronic Dis., Vol. I.), there are three mistakes to be specially guarded against: 1. Fearing lest the doses I have indicated are too small; 2. The improper use of a remedy; 3. Not leaving the dose to exhaust its effects before giving another dose, or changing to another remedy.

Dr. Brewster: The symptoms are a reliable guide to the remedy, but we have as yet no rules as to repetition.

Dr. Nash: When the effect is exhausted?

Dr. Boyce: How are we to know when the effect is exhausted? that is the point.

Dr. Hawley: I have repeated *Lach.* 200, every few hours, then stopped and waited twelve hours, and the case grew worse. The CM repeated every two hours resulted in success. There seems to be no law about repeating; that is, we have not yet discovered one; we are still at sea so far as that is concerned.

Dr. Nash: Dr. Guernsey says we need not fear to repeat until we get some action, then wait until it exhausts itself.

Dr. Hawley: I succeed better in chronic cases by giving but one dose, or three in succession, and then waiting. In acute cases it seems necessary to repeat.

Dr. Seward: There is such variety in constitutions, how are we going to decide? When allopathic medicines are acting perceptibly, give *Nux vom.* If not acting, give what may be indicated by the symptoms present.

Dr. Seward: Symptoms disappear inversely to their appearance. Hence, if a local sore disappear, the internal disease is not therefore shown to be cured. The internal growth of the disease may thereby be increased. We all accept this.

Dr. Boyce: Allopaths act upon this doctrine, and do not treat the external (secondary) sores of syphilis locally.

Dr. Hawley: A young man had a chancre treated topically, and now has soreness in groins, which is painful from exercise and walking; he is otherwise well.

Dr. Boyce: Is it true that numerous chronic diseases are due to the suppression of syphilis, or sycosis, or psora, in their external manifestations?

Dr. Seward: Psora should be thought of in prescribing for chronic diseases; but the remedy is to be selected on the one principle. We cannot treat psora by name.

Dr. Boyce: Hahnemann claimed that remedies not anti-psoric would only palliate, not cure.

Dr. Nash: Hahnemann called a remedy that was successful in curing a chronic malady that was not syphilitic or sycotic, anti-psoric. We may not be able to dispute this, but it seems to be true Homœopathy to give according to the law of similars, covering the totality of the symptoms, without reference to psora.

Dr. Dewel: Are not some of Hahnemann's positions untrue? Can he not be improved on?

Dr. J.: Not in the fundamentals: the law of the similars, the single remedy, and the minimum dose; meaning by minimum dose, that which cures, and does not produce an aggravation. In some other propositions doubtless he erred, and many who are good homœopathists dissent from him in minor matters. Old school physicians succeed, and eclectics also; and mongrels may have even better success than allopaths; but the highest percentage of success is had by pure Hahnemannians.

Dr. Boyce: The appropriate remedy may take effect even if given in alternation; but then, no one can tell which remedy cures. Such a practice cannot progress and will be more or less confused. The inquiry for me now is, what medicine to give. When Hahnemann, failing often to cure with a carefully selected remedy, sought for a long time to find the reason, he found certain remedies more successful in curing chronic diseases, and he came to regard them as especially anti-psoric. At this subject he worked for eleven years.

Dr. Nash: What puzzles me is to know whether he found the anti-psoric to be more homœopathic to the case than the medicine he failed with.

Dr. Nash then read a paper or "Repetition of the Dose," which was accepted and ordered to be published.

Dr. Hawley: Pathology and physiology cannot tell us the processes of disease and life; they are dead.

Dr. Dewel: Pathology and physiology are great helps. Drs. Dunham, Allen and others skilled in Homœopathy used them. We can profit by post-mortem examinations, and in our next case have a better idea of the disease, and of what to do.

Dr. Hawley: The pathological conditions of one case will not enable us to know the exact processes the disease will take in another case. You study pathology on a dead man; whereas in prescribing you have to deal with a live man, not with a dead carcass. There are facts everywhere, but no one can tell the reasons why the facts exist. He must abide by facts, not run after theories. When you know a drug will uniformly produce certain symytoms, and will cure similar symptoms, you have facts. Pathology and physiology are helpful in their facts as to diagnosis and prognosis, but they cannot help in the choice of a remedy.

Dr. Nash: Dr. Lippe once said: "You can't squeeze Homœopathy into that pathological livery; it is too big a thing." Some time ago I had a case of apoplexy; the patient was 80 years of age; one side was completely paralyzed; with the symptom, "sweat as soon as he falls asleep, and even with the closing of the eyes," for an indication, I gave *Conium*, which cured after he had been given up to die. Pathology cannot select the remedy; we must individualize, not generalize.

Dr. Hawley: An allopathic authority says that the chief attention of physicians has been given to diagnosis. It is an important admission for him to make. Another class of men, not noticed by him, have been devoting their time to therapeutics, and I prefer to follow them.

Dr. Nash : There are men who have so much to say concerning pathology, and so plausibly, that many young men are led astray. Because *Bryonia* will remove effusions, therefore, they say, give *Bryonia* in every case of rheumatism where there is effusion. This will not answer.

Dr. Hawley : We must show that true Homœopathy cures more cases than pathological prescribing.

